# Standardized herbal extract PM014 alleviates fine dust-induced lung inflammation in mice

**DOI:** 10.1186/s12906-020-03060-w

**Published:** 2020-09-07

**Authors:** Ye-Seul Lee, Daeun Min, Seon-Young Park, Junyoung Lee, Hyunsu Bae

**Affiliations:** 1 Department of Anatomy and Acupoint, College of Korean Medicine, Gachon University, Seongnam, 13120 South Korea; 2 Department of Physiology, College of Korean Medicine, Kyung Hee University, 26-6 Kyungheedae-ro, Dongdaemoon-gu, Seoul, 02453 Republic of Korea

**Keywords:** pm10, Fine dust, PM014, Chung-sang-Bo-ha-tang, BALF

## Abstract

**Background:**

Fine dust penetrates deep into the human alveoli, and the fine dust accumulated in the bronchus and lungs can directly trigger various respiratory diseases. PM014 (HL301) is the herbal extract derived from the herbal medicine Chung-Sang-Bo-Ha-Tang which is used for the treatment of lung diseases.

**Methods:**

To evaluate the effect of PM014 on the lung inflammation induced by fine dust, this study investigated inflammatory responses in the lung upon pm10 exposure by examining the infiltration of inflammatory cell profiles from bronchial alveolar lavage fluid (BALF), lung histology, and production of pro-inflammatory cytokines measured by RT-PCR and ELISA.

**Results:**

PM014-treated mice exhibited reduced lung tissue damage and inflammatory cell infiltration. Bronchoalveolar lavage fluid (BALF) analysis showed significant decrease in the population of total cells, macrophages, eosinophils, and neutrophils in PM014-treated mice. PM014 treatment downregulated the pro-inflammatory cytokine expressions including IL-1b, IL-8, IL-6, TNF-alpha, IL-21 and IL-17. ELISA analysis also showed reduced production of IL-1b, IL-6 and IL-17 in PM014-treated mice.

**Conclusion:**

PM014 suppressed the pm10-induced inflammatory response in mice. This study shows that PM014 is a possible therapeutic agent for lung inflammation induced by fine dust.

## Background

Fine dust, or Particulate Matter 10 (pm10), is defined as inhalable particles with a diameter of less than 10 μm according to the United States Environmental Protection Agency. These particles vary in size and shape and consist of hundreds of different chemicals. Some are emitted directly from sources such as construction sites, unpaved roads, chimneys, and fires. However, most form in the atmosphere as a result of complex reactions of chemicals such as sulfur dioxide, nitrogen oxides, lead, ozone, carbon monoxide, and pollutants emitted by power plants, industries, and automobiles. It is possible that particle size is linked to the potential for health problems, the biggest problem being that small particles less than 10 μm in diameter can get deep into the lungs and enter the bloodstream. Exposure to these particles can affect both the lungs and the heart. Numerous scientific studies show that particle contamination exposure is associated with a variety of problems such as exacerbation of asthma, decrease in lung function, airway irritation, coughing, and difficulty breathing [1].

PM014 (HL301) is a standardized formulation of Chung-Sang-Bo-Ha-Tang (CSBHT) which has shown to be effective in treating lung diseases through a number of animal studies. Its original formula, CSBHT, is a medication with 18 herbs that has been used in Korea to treat chronic lung diseases such as asthma [2]. As there are 18 herbs in CSBHT, which make it difficult to standardize, the herbal formula has been modified to 7 herbs and standardized into herbal formulation named PM014 (Table 1). Our previous study demonstrates the anti-inflammatory effect of PM014 on radiation-induced pulmonary inflammation in a murine model [3]. In vitro and in vivo results from other studies shows a similar effect of PM014 in murine models of chronic obstructive pulmonary disease (COPD) [4, 5] and cockroach allergen-induced asthma [6]. In addition, PM014 has recently been shown to improve pulmonary fibrosis induced by bleomycin [7]. Furthermore, a thirteen-week study of PM014 showed no subchronic oral toxicity in animal model, providing supportive evidence of the safety of PM014. Taken together, PM014 is a safe and effective herbal formulation which can be applied to chronic and subchronic inflammation in the lungs.

**Table 1:** Components of PM014

Formula of PM014	Amount (g)	Chemical marker
Root of *Rehmannia glutinosa* (RG)	600	5-HMF
Cortex of *Paeonia suffruticosa* (PS)	300	Paeoniflorin
Fruit of *Schizandra chinensis* (SC)	300	Schizandrin
Root of *Asparagus cochinchinensis* (AC)	300	Asparagine
Seed of *Prunus armeniaca* (PA)	225	Amygdalin
Root of *Scutellaria baicalensis* (SB)	225	Baicalin
Root of *Stemona sessilifolia* (SS)	150	Stemonine
Total	2100 g	

From the aforementioned anti-inflammatory effects of PM014 in various models of lung diseases, this study hypothesized that PM014 will alleviate lung inflammation induced by fine dust. We tested this hypothesis by investigating the effect of PM014 on a pm10-induced lung inflammation mouse model. The results showed that PM014 was effective via immunological changes, such as reduced inflammatory cytokines and histopathological changes in the lung tissue in the pm10-induced pulmonary inflammation mouse model.

## Methods

### Animals

Seven-week-old female C57BL/6 mice were purchased from Taconic Korea (DBL, Chungbuk, Korea). All animals were housed in a controlled environment (12/12 h light/dark cycle; 22 ± 1 °C) and had ad libitum access to food and water. This study was approved by the Kyung Hee University Animal Care and Use Committee. All experiments were performed according to regional guidelines established by Kyung Hee University [KHUASP (SE) 19–007].

### Preparation of fine dust

Fine dust (ERM-CZ120) that consisted of arsenic, cadmium, lead, and nickel was purchased from the Institute for Reference Materials and Measurements (Geel, Belgium). This contained 0.5 g of fine dust that was processed to resemble pm10. This was packed into amber glass vials and closed with a rubber stopper and aluminum cap under an argon atmosphere. The mass fraction of the elements of fine dust used in this experiment is presented in Table 2.

**Table 2:** Composition and mass fraction (certified value and uncertainty) of pm10-like fine dust

FINE DUST (pm10-LIKE)		
Element	Mass Fraction	
	Certified value^1)^ [mg/kg]	Uncertainty^2)^ [mg/kg]
Arsenic	7.1	0.7
Cadmium	0.90	0.22
Lead	113	17
Nickel	58	7

^1)^ Unweighted mean value of the means of accepted sets of data, each set being obtained in a different laboratory and/or with a different method. The certified values and their uncertainties are mass fractions based on the mass of the sample after conditioning as described in EN12341 standard of the European Committee for Standardization

^2)^ The certified uncertainty is the expanded uncertainty with a coverage factor *k* = 2 corresponding to a level of confidence of about 95% estimated in accordance with ISO/IEC Guide 98–3, Guide to the Expression of Uncertainty in Measurement (GUM: 1995), ISO, 2008

### Preparation of PM014

The components of PM014 were purchased from Kyung Hee Herb Pharm (Seoul, South Korea) and prepared by Hanlim Pharm Co. LTD (Yongin, Korea) as described in a previous studies [3, 7]. The preparation of the medicinal herbs was based on the standards provided by The Korean Herbal Pharmacopoeia published by the Korean Food and Drugs Administration (KFDA). As *Prunus armeniaca*, of which the seed was used in the preparation of PM014, is classified as endangered by the International Union for Conservation of Nature (IUCN), the use of this plant was limited to minimum for the research purposes only. Process of the preparation of PM014 was done in compliance with the IUCN Policy Statement on Research Involving Species at Risk of Extinction.

Each herb was cut, mixed, dissolved, and extracted using a reflux condenser at 90–100 °C for 3 h and then filtered using a 25-μm sieve. This produced 2100 g of substance according to the ratios indicated in Table 1. Undesirable materials were removed by filtration (Sigma, St. Louis, MO, USA). The solvent was concentrated at 60 °C using an evaporator under vacuum, and the final product of PM014 was collected in the form of dried extract powder [7]. PM014 (20 mg/ml) was dissolved in high-performance liquid chromatography (HPLC)-grade methanol and filtered using Minisart RC15 (Sartorius Stedim Biotech, Germany). In the experiment, PM014 was further diluted for administration using phosphate-buffered saline (PBS) at different concentrations. In the final PM014 extract, the quantities of standard materials per 1 g were paeoniflorin > 0.43 mg, schizandrin > 0.12 mg, baicalin > 7.26 mg, and amygdalin > 2.48 mg. High-performance liquid chromatography analysis was performed to quantify the standard materials of PM014. Three independent data were produced for the analysis from three independent batches of each compound. The standardized formula of PM014 was approved by the Ministry of Food and Drug Safety, Republic of Korea for the Investigational New Drug (IND) program (ID: 20130030575). The voucher specimen of this material has been deposited in Hanlim Pharm, Co. LTD, and the deposition number is PURI 15004.

### Fine dust (pm10)-induced lung inflammation mouse model

Fine dust (100 μg/ea) was used to induce lung inflammation in mice. Female C57BL/6 mice were randomly divided into six groups: control, pm10 group, pm10 + PM014 group, and pm10 + DEXA (dexamethasone, 10 mg/kg). The most efficient concentration of PM014 to elicit inhibitory effects on lung inflammation was investigated in our previous study to be a dose of 200 mg/kg [3]. For the evaluation of different doses used in this study those administered therapeutically, we referred to the guidance for industry prepared by the Office of New Drugs in the Center for Drug Evaluation and Research at the Food and Drug Administration [3, 8]. The optimal timing for the administration of PM014 was applied based on another preliminary experiment [7], and we decided to use continual treatment of PM014 to examine the significant impact on fine dust-induced inflammation. Both PM014 and DEXA were administered orally (peroral administration, p.o.).

In the pm10 + PM014 group, the dose of PM014 were different across three subgroups: 50, 100, or 200 mg/kg. On days 0, 2, 7, and 9, mice were lightly anesthetized with isoflurane and tested for a prophylactic effect by intratracheal administration of pm10 dissolved in 50 μl of PBS 2 h after oral administration of PM014. On days 5 and 12, only PM014 was administered without injecting fine dust. pm10 was given twice a week and PM014 was given three times a week. On day 14, all mice were euthanized and harvested to analyze the lungs.

### Analysis of bronchoalveolar lavage fluid

During intrathecal (IT) injection, wet paper towel with isoflurane (100 ul) was placed underneath the wire mesh in the anesthesia glass jar (500 ml) to prevent direct contact of animal with the isoflurane. Mouse was placed in the jar for 1 min with the lid closed. Euthanasia was performed with mice placed in the glass jar (500 ml) with an overdose isoflurane (250 ul) per mouse for 5 min. During euthanasia, mice were constantly observed to check their breathing. After all of the animals were euthanized, bronchial alveolar lavage fluid (BALF) was collected by washing the lungs three times with 1 ml of PBS. BALF samples were centrifuged at 2000 rpm for 10 min at 4 °C and the cell pellet was resuspended in 1 ml of PBS [7]. A hemocytometer was used to count the total number of live immune cells. The cells were then cytospun onto microscope slides and stained with the Diff-Quick Staining Kit (Thermo Fisher Scientific, Waltham, MA, USA). Cells (500 per slide) were counted under an optical microscope [3, 7].

### Histopathological staining

The left lung was isolated from the mouse and immediately fixed with 4% paraformaldehyde before serial tissue processing and embedding with paraffin wax. For histological studies, tissues were cut into 5 μm sections using a microtome, and deparaffinized tissue sections were stained with eosin and hematoxylin (H&E, Sigma, St. Louis, MO, USA) before mounting to evaluate the morphological changes in the lungs.

### Quantitative real-time PCR

Total RNA was extracted from lung tissues and cells using Easy Blue™ (Intron Company, Seongnam, Korea). The first-strand cDNA was synthesized using a cDNA synthesis kit (Bioneer Corporation, Daejeon, Korea). Subsequently, mRNA levels of tumor necrosis factor-α (TNF-α), interleukin (IL)-1β, IL-6, IL-8, interferon-γ (IFN-γ), monocyte chemoattractant protein 1 (MCP-1), IL-17, IL-21, and IL-23 were determined using LightCycler 96 (Roche, Basel, Switzerland) along with SYBR Green qPCR Mastermix (Bioline Reagents Ltd., London, United Kingdom). Relative levels of mRNA expression in 20 μl were normalized to the expression of β-actin for each gene.

### ELISA

Protein concentration of the lung was determined using the albumin standard (Thermo Fisher Scientific, Waltham, MN, U.S.A). To measure cytokine concentration of lungs, sandwich enzyme-linked immunosorbent assay (ELISA) was conducted. IL-1b, IL-6, and IL-17 DuoSet ELISA (R&D System, Minneapolis, MN, U.S.A) were used in this experiment according to the manufacturer’s protocol. Optical density was measured at 450 nm by using a microplate reader.

### Statistical analysis

Statistical analysis was performed using Prism 5 software (Graph Pad Software Inc., San Diego, CA, USA). Data were represented as means ± SEM. A one-way analysis of variance (ANOVA), followed by the Newman-Keuls test, was performed. *P* < 0.05 was considered statistically significant.

## Results

### Effect of PM014 on pm10-induced histological changes in lung tissue

**Fig. 1:**
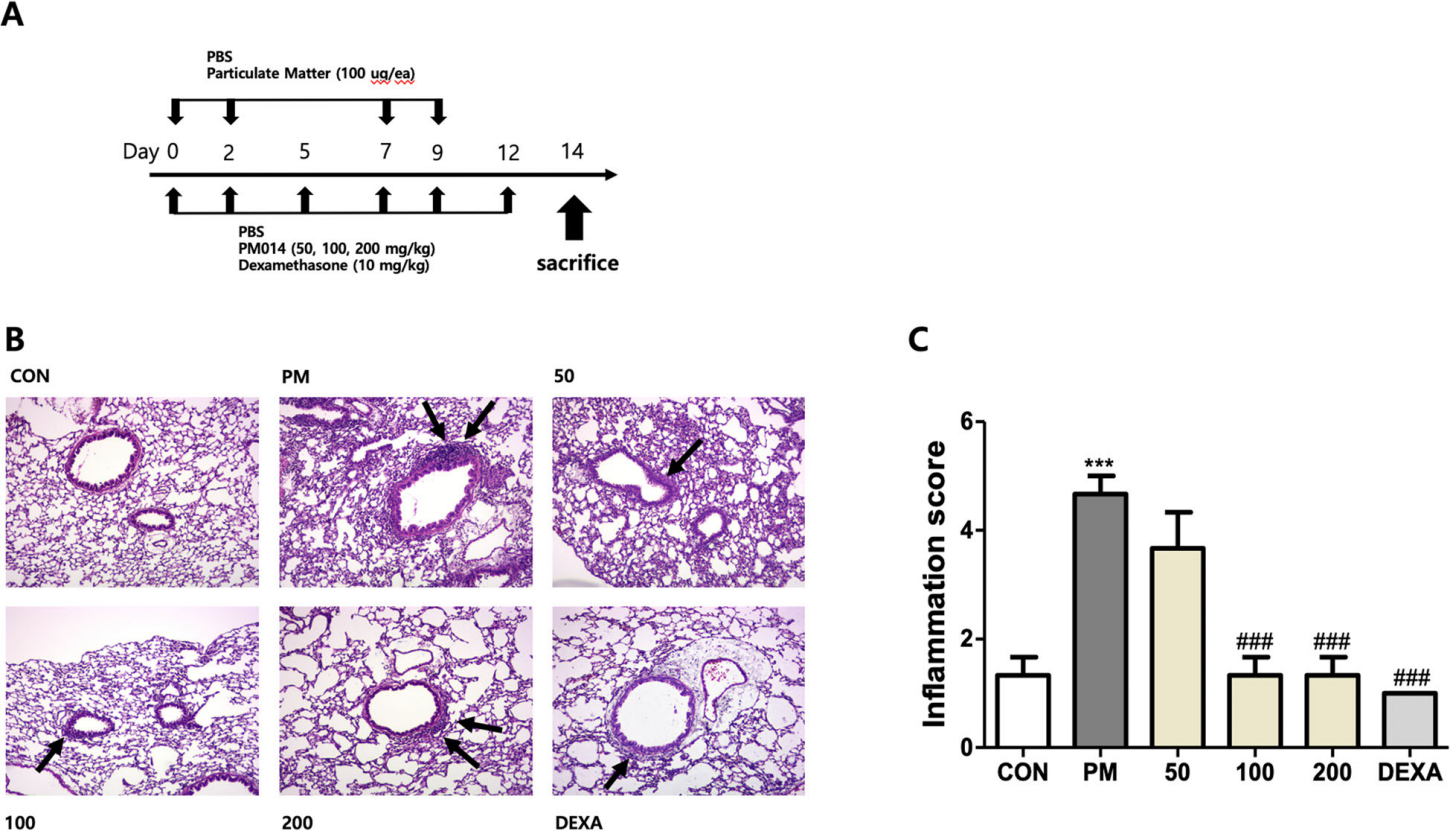
The effects of PM014 on histopathological changes in lung tissues Control, phosphate buffered saline (PBS)-treated; PM, pm10 + PBS; 50, pm10 + 50 mg/kg PM014; 100, pm10 + 100 mg/kg PM014; 200, pm10 + 200 mg/kg PM014; and DEXA, pm10 + 10 mg/kg dexamethasone. **a** The experimental schedule was represented. **b** Lung tissues were stained with H&E to observe inflammatory cell infiltration. Infiltrated immune cells were marked with arrows. Magnification, 20x. **c** Quantification of inflammatory foci. Data are expressed as mean ± standard error (***P < 0.001 versus control; ###*P* < 0.001, versus PM + PBS; *n* = 7–10)

Histopathological changes and inflammatory responses due to pm10-induced lung injury in PM-treated mice showed the accumulation of multiple inflammatory cells in alveolar spaces after 2 weeks. Alveolar infiltration by inflammatory cells in the PM group was significantly higher than that in the control group. However, PM014-treated mice showed reduced tissue damage, which included less inflammatory cell infiltration in the alveoli (Fig. 1a and b), compared to the control group.

### Effect of PM014 on inflammatory cell infiltration in the lungs

**Fig. 2:**
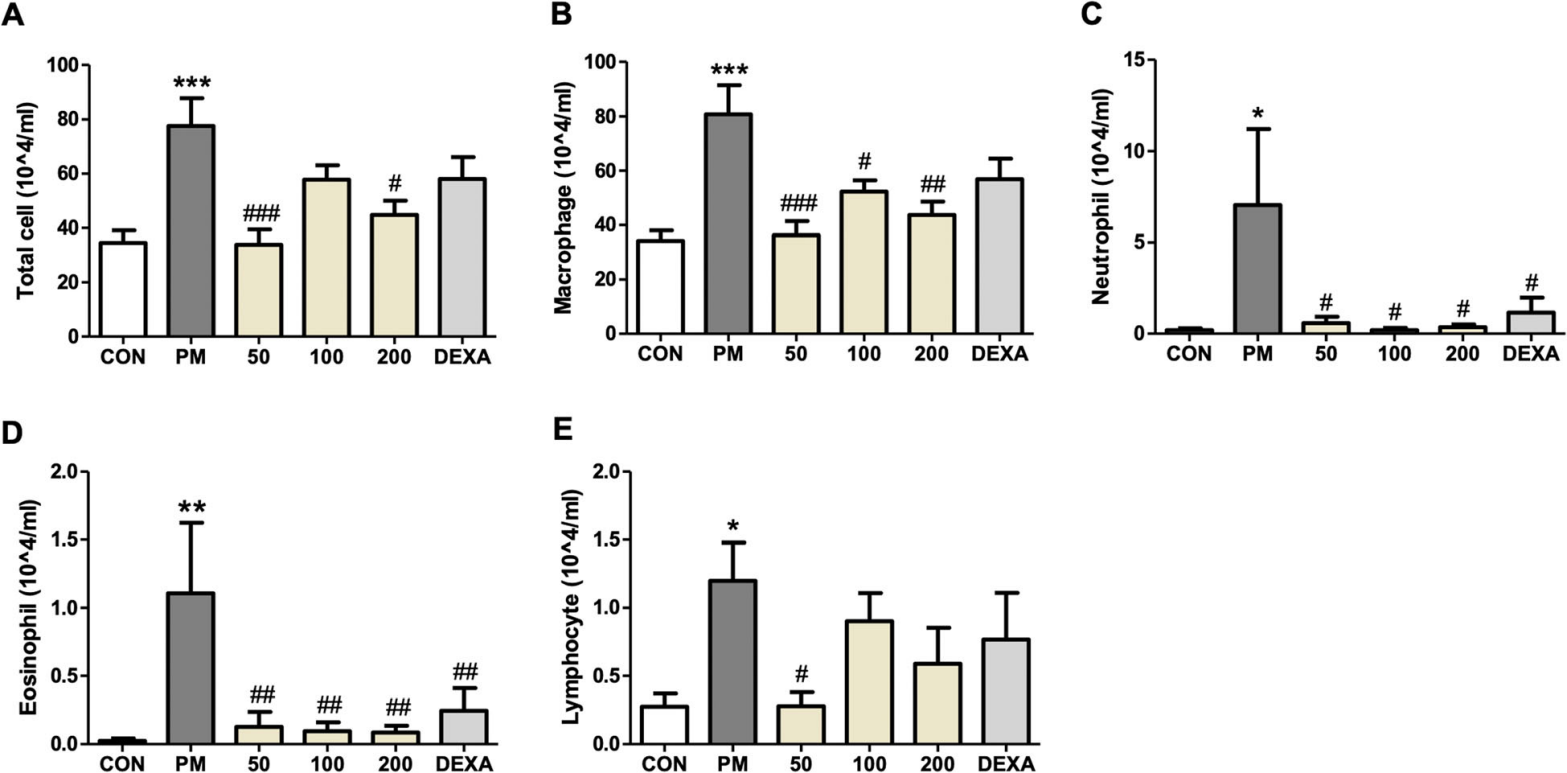
Effect of PM014 on infiltration of immune cells in bronchoalveolar lavage fluid (BALF). Differential cell count of BALF: total cell count (**a**), macrophages (**b**), neutrophils **(c)**, eosinophils **(d)**, and lymphocytes **(e)**. Control, phosphate buffered saline (PBS)-treated; PM, pm10 + PBS; 50, pm10 + 50 mg/kg PM014; 100, pm10 + 100 mg/kg PM014; 200, pm10 + 200 mg/kg PM014; and DEXA, pm10 + 10 mg/kg dexamethasone. Data are expressed as mean number of cells ± standard error (*P < 0.05, **P < 0.01, and ***P < 0.001 versus control; #*P* < 0.05, ##*P* < 0.01, and ###*P* < 0.001 versus pm10 + PBS; *n* = 7–10)

The total cell count of BALF in the PM group was significantly higher than that in the control group (Fig. 2a). In the PM group, macrophages (Fig. 2b), neutrophils (Fig. 2c), eosinophils (Fig. 2d), and lymphocytes (Fig. 2e) were significantly higher than in the control group. In contrast, the population of total cells, macrophages, neutrophils, and eosinophils in PM014-treated mice was significantly lower than that in PM-treated mice.

### Effect of PM014 on inflammation-related gene expression in the lungs

**Fig. 3:**
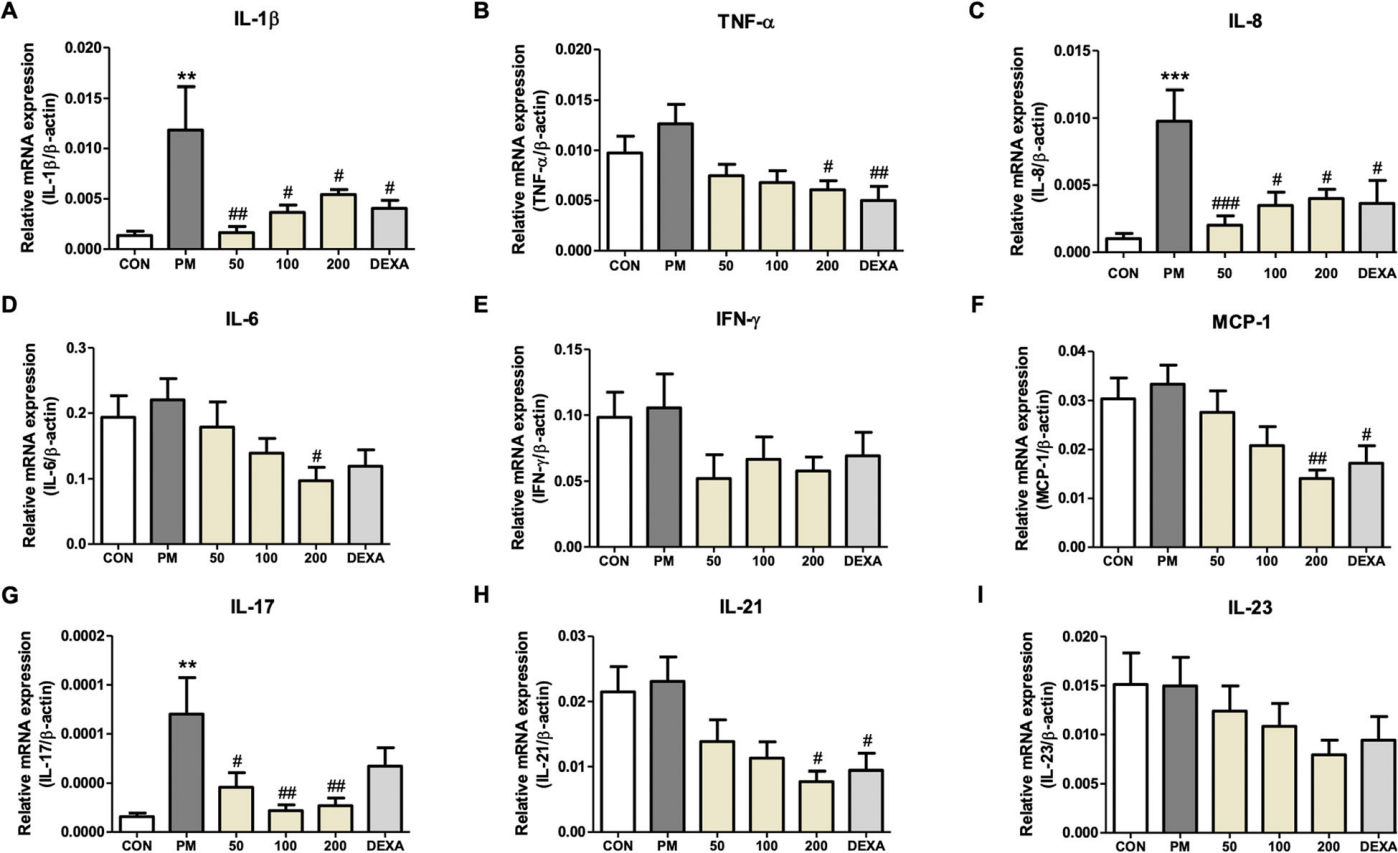
Effect of PM014 on relative mRNA expression in the lungs. Control, phosphate buffered saline (PBS)-treated; PM, pm10 + PBS; 50, pm10 + 50 mg/kg PM014; 100, pm10 + 100 mg/kg PM014; 200, pm10 + 200 mg/kg PM014; and DEXA, pm10 + 10 mg/kg dexamethasone. IL-1b **(a)**, TNF **(b)**, IL-6 **(c)**, IL-8 **(d)**, IFN-g **(e)**, MCP-1 **(f)**, IL-17 **(g)**, IL-21 **(h)**, and IL-23 **(i)**. The ratio of mRNA to ±-actin was depicted as mean ± standard error (**P < 0.01 and ***P < 0.001 versus control; #*P* < 0.05, ##*P* < 0.01, and ###*P* < 0.001 versus pm10 + PBS; *n* = 7–10)

The expression of cytokines IL-1b, IL-8, and IL-17 (Fig. 3a, c, and g) was significantly higher in the PM-treated mice than that in the control group. However, treatment with PM014 reduced the expression of these cytokines. The rest of the cytokines and chemokines did not increase significantly in the PM-treated mice, but the levels of these cytokines and chemokines in the group treated with PM014 showed a tendency to decrease in comparison to the PM group (Fig. 3b, d–f, h, and i).

### Effect of PM014 on inflammation-related cytokine production in the lungs

**Fig. 4:**
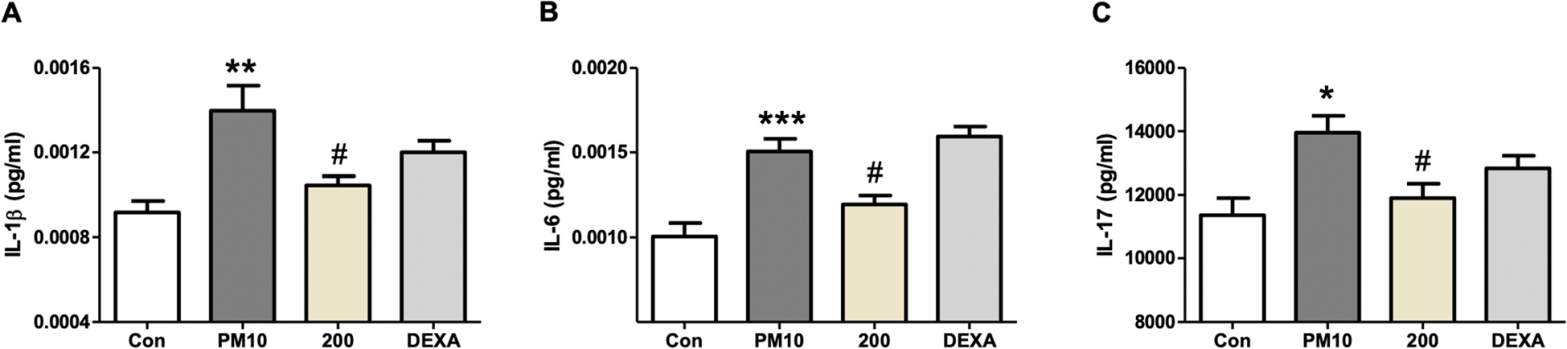
Effect of PM014 on cytokine production in the lung. Control, phosphate buffered saline (PBS)-treated; PM, pm10 + PBS; 200, pm10 + 200 mg/kg PM014; and DEXA, pm10 + 10 mg/kg dexamethasone. IL-1b (**a**), IL-6 (**b**) and IL-17 (**c**). Data are expressed as mean ± standard error (*P < 0.05, **P < 0.01 and ***P < 0.001 versus control; #*P* < 0.05 versus pm10 + PBS; *n* = 7–10)

ELISA was used to measure inflammation-related cytokine production of lungs. In Fig. 4(a-c), the amount of IL-1b, IL-6, and IL-17 was significantly higher in the PM-treated mice than that in the control group. DEXA group showed a tendency to decrease in comparison to the PM-treated group. However, treatment with 200 mg/kg PM014 reduced significantly amount of these cytokines.

## Discussion

pm10, a type of fine dust with inhalable particles and typically less than 10 μm in diameter, is increasingly diffusing into the atmosphere and emerging as a threat to the health of those who breathe in air with pm10 [1]. The related health problems are mainly due to its small size, specifically because particles less than 10 μm in diameter can go deep into the lungs and into the bloodstream [9]. Exposure to pm10 can affect both the lungs and the heart, which causes inflammation in the lungs and cardiac rhythm abnormalities. A number of previous studies show that particle contamination exposure is associated with a variety of problems such as exacerbation of asthma, decrease in lung function, airway irritation, coughing, and difficulty breathing [10–13]. Therefore, this study investigated how PM014, a herbal formula that has previously been shown to relieve symptoms related to the lungs such as asthma and COPD [2–4, 7], affects fine dust-induced lung inflammation in a fine dust-induced acute lung injury mouse model. This preventive effect of PM014 on fine dust-related diseases may have an important implication in the future as the impact of fine dust is becoming both substantial and unavoidable.

CSBHT is a herbal formula well-known for the treatment of lung diseases in Korean Medicine, and PM014 in this study is a standardized herbal formula with seven herbs extracted from CSBHT [6]. We had investigated the effect of PM014 in comparison with the effects of individual ingredient herbs using an acute lipopolysaccharide-induced lung injury model, which showed a greater reduction of immune cell infiltration in the lungs with the treatment of PM014 [3, 4]. In this study, the reduction of immune cell infiltration in the lungs due to PM014 was greater than that from treatment with individual herbs. A preliminary experiment which aimed to identify the optimal dosage of PM014 was performed with different doses of 50, 100, 200, and 300 mg/kg, and the result showed that 200 mg/kg is the most efficient [3]. In this study, we referred to the guidance for industry prepared by the Office of New Drugs in the Center for Drug Evaluation and Research at the Food and Drug Administration for the evaluation of the doses in comparison with the doses that were administered therapeutically [3, 8]. According to this guidance, treatment with 200 mg/kg of PM014 in a mouse model implies that 1 g of PM014 is equivalent to the dose in a 60 kg human. Furthermore, another preliminary experiment was performed to discover the optimal timing for the administration of PM014, in which a single treatment for 1 week and continual treatment for three times a week of PM014 was compared [7]. This experiment showed that treatment once a week did not have significant effects; therefore, we decided to use continual treatment, rather than a single treatment, of PM014 to examine the significant impact on fine dust-induced inflammation.

Although the mechanism of the lung inflammatory response from pm10 is unclear, studies on the impact of pm10 on lung inflammation show decrease in microvascular function and increase in leukocyte, neutrophil, and eosinophil counts [14]. We previously reported the mechanism of the protective effects in the lungs exerted by PM014, which in vitro showed that PM014 led to the inhibition of TGF-β1-induced epithelial-mesenchymal transition (EMT) and fibroblast activation in alveolar epithelial cells and human lung fibroblasts [3, 7]. This study showed that TGF-β1 signaling via p38 mitogen-activated protein kinases (MAPKs) pathways and Smad-dependent pathways were the main targets of PM014. Furthermore, PM014 treatment led to the downregulation of inflammatory cytokines, chemokines, and fibrosis-related genes, and reduction in the growth of factor-β1-positive cell population in lung tissue [3, 7, 15].

Our study showed a consistent result with respect to the influence of pm10 on lung function and showed an increased level of macrophages, neutrophils, eosinophils, and lymphocytes. Furthermore, in our study, pm10 induced increased levels of IL-1β, IL-8, and IL-17. Previous studies show that IL-8 dependent inflammation implies innate immunity of the lung to bacterial pathogens, as well as chronic lung inflammation and impaired function that are progressive and irreversible, such as chronic obstructive pulmonary disease (COPD), bronchiectasis, and pulmonary fibrosis [16]. An elevated level of IL-1β indicates pulmonary inflammation, emphysema, and airway remodeling and is increased in the lungs of patients with COPD and asthma [17]. Lastly, IL-17 promotes skin and lung inflammation [18]. Overall, in our study, pm10 caused lung inflammation in mice that led to chronic and irreversible changes in lung function.

This study assessed the effect of PM014 on tissue inflammatory responses, in specific on the immune cell infiltration and cytokine expression, 2 weeks after the start of the experiment. Although fine dust, represented by pm10 in our study, caused lung inflammation, PM014 treatment led to a significant decrease in immune cell infiltration around the bronchioles (Figs. 1 and 2). During inflammatory reactions, inflammatory cells including macrophages, neutrophils, fibroblasts, and lymphocytes are activated, leading to the release of inflammatory and fibrotic cytokines [19]. Our results showed that PM014 treatment significantly reduced inflammatory cell infiltration in BALF (Fig. 2), which implied a significant reduction in the population of macrophages. These results suggest that the main attribute of the anti-inflammatory effect of PM014 may be the inhibition of the activation of macrophages.

Furthermore, our discoveries are consistent with an earlier report in which PM014 blocked the inflammatory cells influx and decreased pro-inflammatory cytokines in mice [4, 7]. In this study, exposure to pm10 increased the inflammatory cells infiltration and production of pro-inflammatory cytokines on day 14. Pro-inflammatory cytokines including IL-8, IL-1β, and IL-17 were released from the inflammatory cells. On the other hand, daily administration of PM014 was proven to be effective in the prevention of the secretion of cytokines due to pm10 [7]. No significant difference was observed in the expression levels of other cytokines such as IL-6, IL-21, IL-23, and TNF between pm10 mice and control mice. IL-23 acts as a regulator that promotes the development of IL-17-producing helper T cells [20]. In addition to this knowledge, recent studies show that not only the Th17 cells, but also other cells such as CD8^+^ T cells and neutrophils produce IL-17 [21], which calls for a further investigation on the role of T cells in the immune responses in the lungs. Overall, our results suggest that PM014 alleviates lung inflammation induced by pm10 via reduction of pro-inflammatory cytokines, which are produced by the inflammatory cells. Future studies that explore how PM014 directly plays the role of an anti-inflammatory substance in lung inflammation due to pm10 may provide further information on the mechanism of the therapeutic effects of PM014.

## Conclusion

Fine dust is an air pollutant that contains chemicals such as sulfuric acid gas and nitrogen oxides that are generated in power plants, automobiles, and factories. Fine dust, also known as PM, penetrates deep into the human alveoli and the fine dust accumulated in the bronchus, and lungs can directly trigger various respiratory diseases and reduce immune function. PM014 is a herbal extract derived from the herbal medicine CSBHT, which is used for the treatment of lung diseases in traditional medicine. In this study, we observed that fine dust increased the infiltration of inflammatory cells in the lungs of mice. The expression of pro-inflammatory cytokines was measured by RT-PCR and PM014-treated mice exhibited reduced tissue damage, inflammatory cell infiltration, and pro-inflammatory cytokine gene expression. Our study showed that the administration of PM014 suppressed the pm10-induced inflammatory response in mice.

## Data Availability

The datasets used and/or analysed during the current study are available from the corresponding author on reasonable request.

## Abbreviations

pm10: Particulate Matter 10
BALF: Bronchial alveolar lavage fluid
CSBHT: Chung-Sang-Bo-Ha-Tang
COPD: Chronic obstructive pulmonary disease
PBS: Phosphate buffered saline
